# Same-same, but different? Postoperative rhythm disturbances and other analysis after rotational Z-flap vs. patch repair of sinus venosus atrial defects

**DOI:** 10.3389/fcvm.2026.1841248

**Published:** 2026-06-17

**Authors:** Raphael Groß, Melika Hajymiri, Mohamed Elbayomi, Frank Harig, Friedrich Mellert, Robert Cesnjevar, Presheet Pathare

**Affiliations:** 1 Department of Cardiac Surgery, Friedrich-Alexander-University, Erlangen, Bavaria, Germany; 2 Latner Thoracic Surgery Research Laboratories, University Health Network and University of Toronto, Toronto, ON, Canada

**Keywords:** atrial septal defect, congenital heart diseases, postoperative rhythm disturbances, rotational Z-flap, sinus venosus atrial defect

## Abstract

**Background and aims:**

Surgical correction of sinus venosus atrial septal defects (SVASD), often associated with partial anomalous pulmonary venous return (PAPVR), is characterized by low mortality but carries a risk of postoperative rhythm disturbances due to the proximity of the sinus node. This study aimed to compare the clinical outcomes and incidence of postoperative arrhythmias between two surgical techniques: the conventional patch repair (baffle) and the rotational “Z” flap technique.

**Methods:**

A retrospective, single-center non-inferiority analysis was conducted on 110 patients (adult and pediatric) who underwent isolated SVASD correction between 2000 and 2025 in northern Bavaria, Germany. Patients with preoperative arrhythmias or concomitant procedures were excluded. Subjects were divided into two cohorts: the baffle group (*n* = 70) and the “Z” flap group (*n* = 40). The primary endpoint was documented postoperative rhythm disturbance at 3–6 months follow-up on EKG. Secondary endpoints included operative times, residual shunts, and SVC (Superior vena cava) stenosis.

**Results:**

Operative times were significantly shorter in the “Z” flap group compared to the baffle group (Total time: 182.6 vs. 209 min; Bypass time: 71.4 vs. 96.1 min; Cross-clamp time: 35.7 vs. 46 min; all *p* < 0.05). Postoperative rhythm disturbances occurred in 54.5% of the total population, with the right bundle branch block (40.1%) being the most frequent. Notably, the “Z” flap group showed a significantly higher incidence of sinoatrial block with atrio-ventricular junctional rhythm (17.5% vs. 4.3%; *p* = 0.035; OR = 4.47). No patients required permanent pacemaker implantation, and no significant differences were found regarding residual shunts or superior vena cava stenosis.

**Conclusion:**

Both surgical methods are effective and contain inherent advantages and disadvantages for SVASD correction. While the “Z” flap technique is surgically more efficient with shorter ischemic times, it is associated with a significantly higher risk of developing postoperative junctional rhythms compared to the conventional baffle repair.

## Introduction

If a perfect method to operate a patient existed, we could all stop writing about surgery and focus on performing it. Unfortunately, this is not the case. Since multiple operative methods using the same principle exist for treating the sinus venous defect, we thought it would be prudent to attempt to find out if they differentiate themselves only in technique, or also in the outcome. The “Z” flap technique gained popularity due to the avoidance of incision across the Cavo atrial junction, thereby preserving the sinus node and its blood supply. By contrast, conventional baffle repairs (single-patch or two-patch) require incisions at or near the SVC (Superior vena cava)–right atrial junction, directly threatening the sinus node. Reviewing the current literature shows that operative therapy for sinus venous defects is associated with an extremely low morbidity and mortality, and no significant difference in the incidence of postoperative rhythm disturbances. These results form the basis of the assumption of non-inferiority between the techniques. With the results available in current literature, we decided to conduct a retrospective analysis after rotational Z-flap vs. patch repair of Sinus venosus atrial defects with a focus on the occurrence of postoperative rhythm disturbances.

## Patients and methods

### Study design and patient population

The study hypothesized that both methods of correction had a similar rate of postoperative rhythm disturbances. Considering the low expected morbidity and mortality of the therapy, our null hypothesis was one of non-inferiority.

We undertook a single-center retrospective cohort study using our cardiothoracic database and electronic patient records at our university hospital in northern Bavaria, Germany, from the year 2000 through 2025. All patients, adult and pediatric, undergoing an isolated sinus venosus atrial defect correction were included in the analysis. After excluding patients with documented preoperative rhythm disturbances, patients undergoing concomitant procedures, redo procedures, and missing records; 110 patients were included in the initial analysis.

Inclusion criteria:

- All patients, adult and pediatric, undergoing sinus venosus atrial defect correction.

Patients were then excluded based on the following criteria.

- Patients with preoperative rhythm disturbances or a history of an intervention for rhythm disturbances (including, but not limited to: pacemaker implantation, pulmonary vein ablation, etc.)
- Redo operations
- Patients receiving concomitant procedures/corrections (e.g., valve replacement/repair, correction of other congenital heart diseases, CABG etc.)
- Patients who had preoperative rhythm disturbances were also to be excluded.

The 110 patients who underwent an isolated sinus venosus atrial defect correction were subdivided into two categories depending on the technique used to reconstruct the superior vena cava. An interesting trend noted during the analysis was the absence of patients with preoperative rhythm disturbances, after applying the first three exclusion criteria.

The primary endpoint of this study was defined as documented persistent postoperative rhythm disturbance at follow up (usually 3–6 months postoperatively) on ECG.

Secondary analysis was conducted using operative times, postoperative cardiac function, presence of rest shunt, causes for revision and echocardiographic assessment of the superior vena cava and pulmonary vein.

### Operative technique

**Figure 1:**
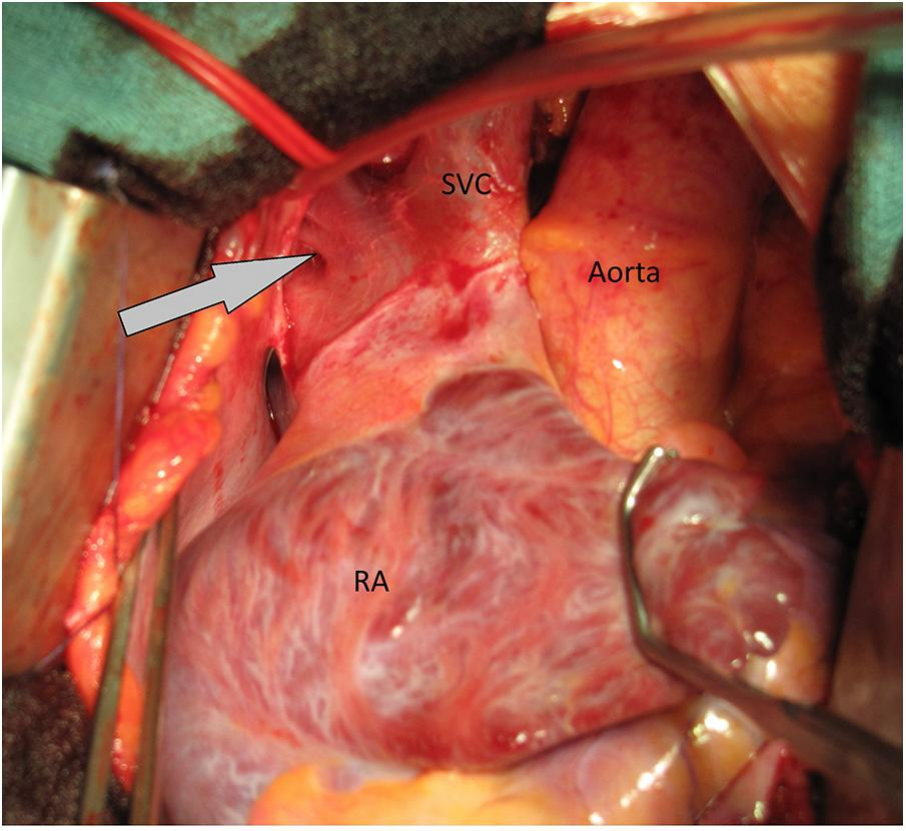
Sinus venous defect. Figure showing a partial anomalous pulmonary venous return (PAPVR) into the superior vena cava (gray arrow) in a case of sinus venosus defect after opening the pericardium. SVC, superior vena cava; RA, right atrium.

**Figure 2:**
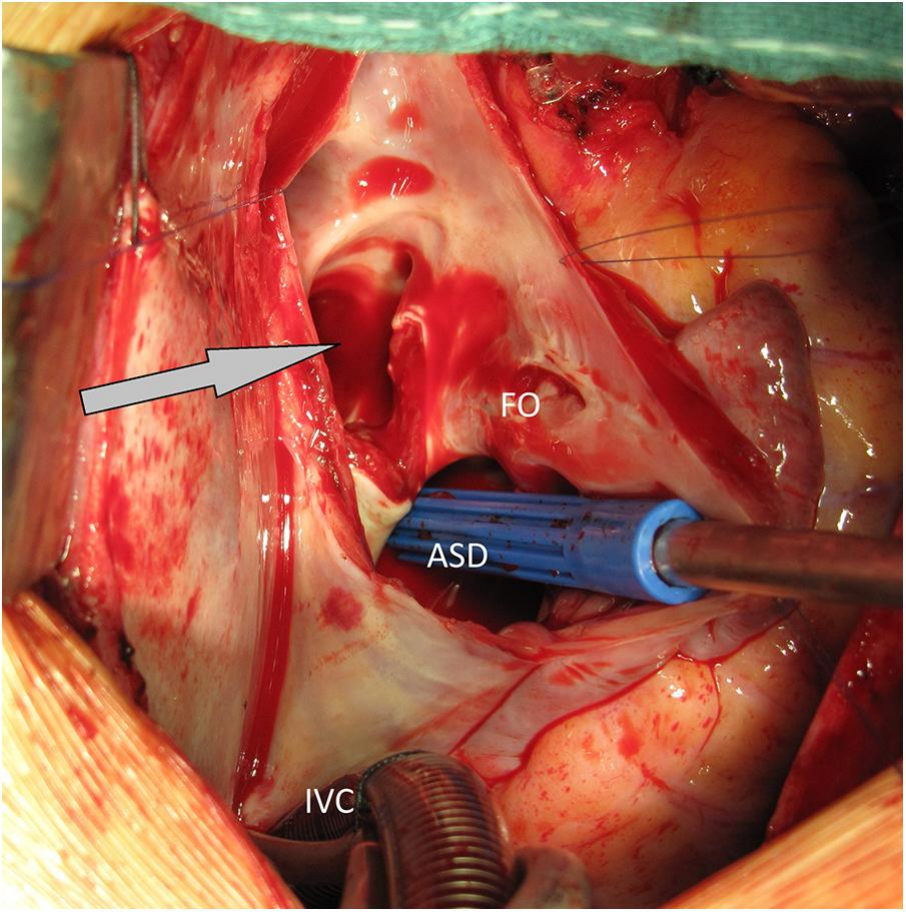
Atrial septal defect. Suction placed through the atrial septal defect in a case of sinus venosus defect after opening the right atrium. Partial anomalous pulmonary venous return (PAPVR) (gray arrow). FO, Foramen ovale; ASD, atrial septal defect; IVC, inferior vena cava cannula.

**Figure 3:**
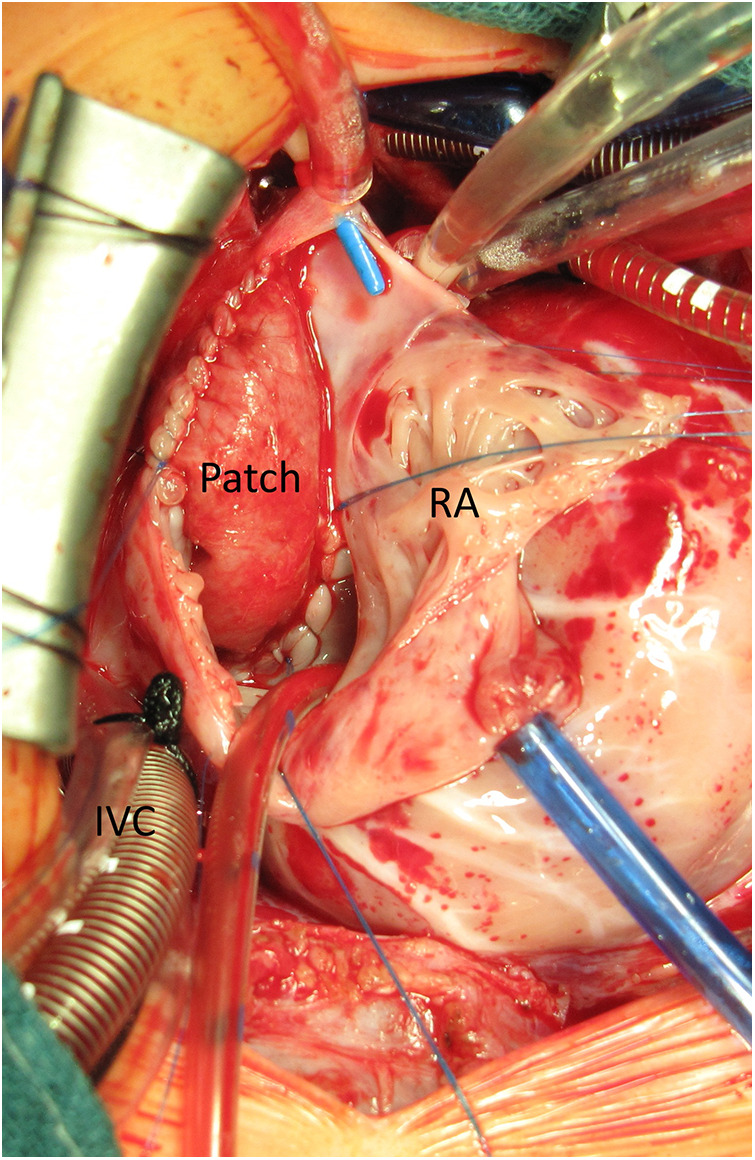
Atrial septal defect correction. Correction of the atrial septal defect and redirection of blood flow of the aberrant pulmonary vein using bovine pericardial patch in a case of sinus venosus defect. RA, right atrium; IVC, inferior vena cava cannula.

**Figure 4:**
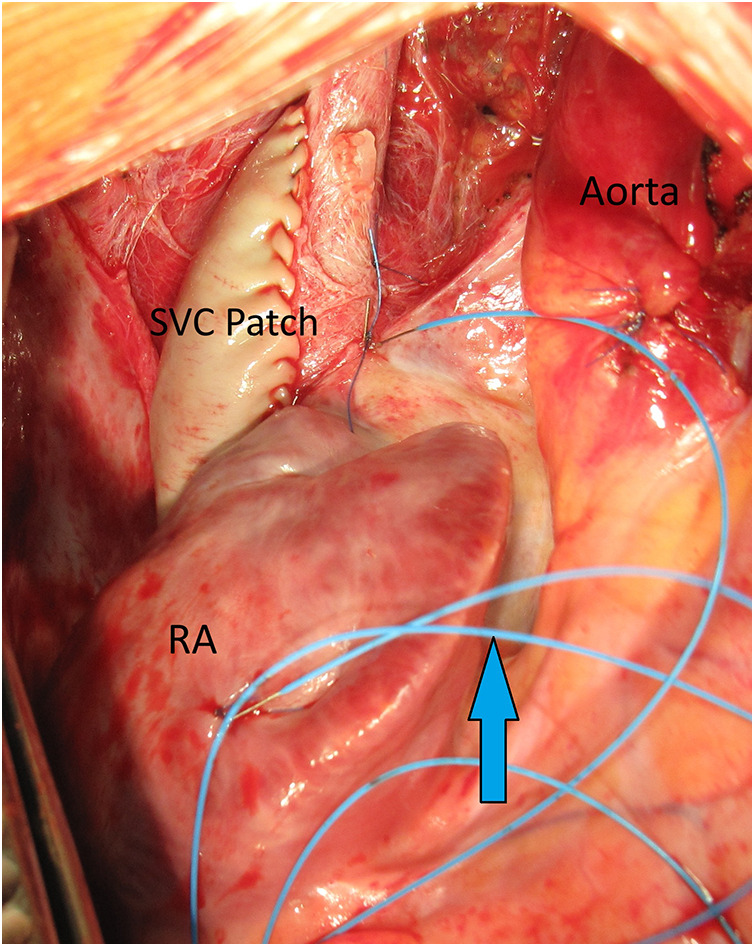
Reconstruction of the superior vena cava via baffle. Reconstruction of the superior vena cava using a bovine pericardial patch. SVC, superior vena cava; RA, right atrium. Blue arrow-temporary pacemaker wires.

**Figure 5:**
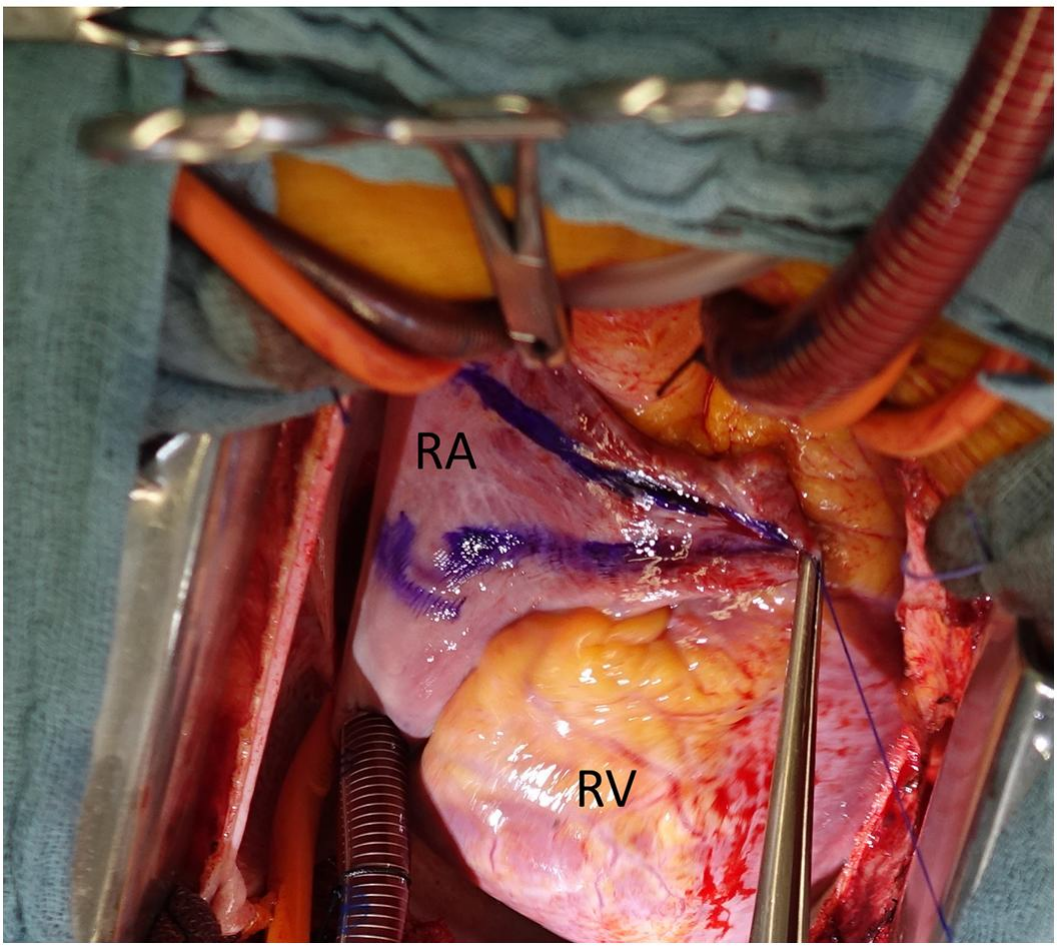
Preoperative marking of the rotational “Z” flap. Marking on the right atrium of the rotational flap, to be used in the reconstruction of the superior vena cava. RA, right atrium; RV, right ventricle.

**Figure 6:**
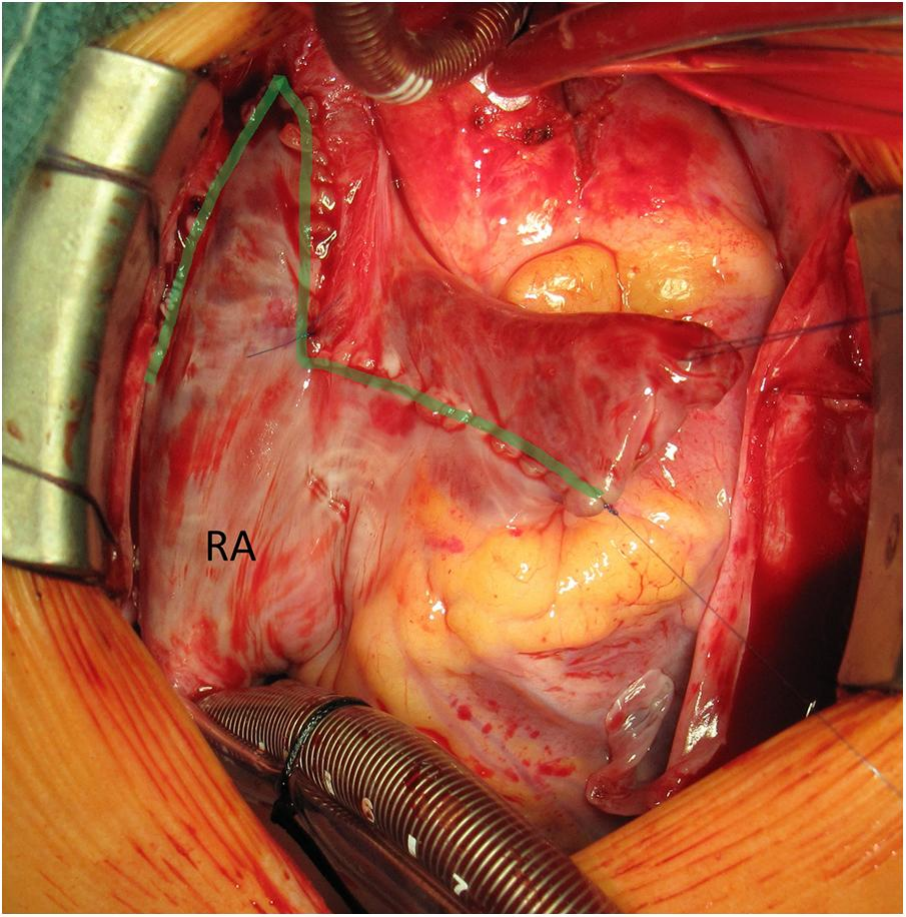
Postoperative superior vena cava reconstruction. Postoperative result of superior vena cava reconstruction using the “Z” flap. RA, right atrium. Green marking-suture line of the rotational “Z” flap.

All patients underwent a complete sternotomy. After incision of the pericardium, the diagnosis of sinus venosus atrial defect with a partial anomalous pulmonary venous return (PAPVR) (Figure 1) and an atrial septal defect (Figure 2) is confirmed. Cannulation for cardiopulmonary bypass was performed using selective cannulation of the superior and inferior venae cavae, along with the ascending aorta. The superior vena cava cannulation was performed cephalad to the points of entry of the branches of the right superior pulmonary vein. Crossclamping of the aorta was performed, following which cardioplegia was administered via a needle vent in the aortic root. The repair of the atrial septal defect was performed using a bovine or glutaraldehyde fixed autologous pericardial patch where available (Figure 3). The patch was also used to divert the blood flow of the anomalous pulmonary vein into the left atrium. The atriotomy incision location relative to the cavoatrial junction being the determinant of nodal injury, was taken as far away from the anatomical location of the Sino-Atrial Node. Reconstruction of the superior vena cava was performed using one of two methods: A baffle using bovine pericardium, (Figure 4) or a preoperatively prepared rotational “Z” flap (Figure 5), of the right atrium, as shown in Figure 6. Factors affecting the selection of technique included, but were not limited to: relative size of the right atrium, preference of the surgeon, and anatomy of the defect. For e.g. larger right atrium size favored the use of Z-Flap, while smaller anatomy favored the use of a double patch technique. Care was taken to at closure of the right atrium ensure tension free suturing. Deairing was performed using a catheter placed in the right upper pulmonary vein and the aforementioned needle vent. Subsequent decannulation was performed after successful weaning off the cardiopulmonary bypass.

#### Human participant protection

This retrospective study complied with the Helsinki Declaration (2000). Authorization for this specific analysis was granted by the local Ethics Committee (EC, No. 26-225-Br), predicated on the retrospective retrieval of existing patient data. The EC waived the need for further written patients' informed consent.

#### Data collection and statistical analysis

All patient-associated data were collected in an approved manner on a comprehensive spreadsheet. The Null hypothesis was one of non-inferiority, or specifically, that no method was presumed to be better than the other. Continuous variables are presented as mean ± SD median and interquartile range (IQR), as appropriate. Categorical variables are presented as frequencies and percentages. Comparisons between groups were performed using Welch's *T*-Test and two-tailed test for continuous variables and the Fisher's Exact Test for categorical variables, as appropriate. All statistical tests were two-sided, and a *p*-value <.05, was considered statistically significant. Statistical analyses were performed using Stata/SE statistical software version 17.0 (StataCorp.; College Station, TX, USA). No specific *a priori* hypotheses were defined for subgroup analyses, which should therefore be considered exploratory.

## Results

With respect to baseline characteristics of the 110 patients, there were 47 females and 63 males. The average age of the patients was 15.2 (SD ± 20.7) years with a range of 0.5–83 years. The average weight was 33.2 kg (SD ± 27.1) with a range of 3.9–100. Average height was 123.2 cm (SD ± 36.6) with a range of 55–183 cm. The patients were then further divided into the respective cohorts. There were 70 patients who were placed in the baffle cohort, and 40 patients were placed in the cohort undergoing modified reconstruction using “Z” flap technique. Due to the large range disparity in age, size and weight, there were, as mentioned above, a large number of older adults who underwent repair using Z flap, and a significant number of younger patients undergoing correction using a double patch technique. Detailed baseline characteristics of patients after subdivision into cohorts can be found in Table 1.

**Table 1:** Baseline patient characteristics^Ψ^.

Characteristic	Total	Correction by Baffle	Correction by. “Z” flap.	P value
Age of Patient – yr	15.2 ± 20.7	10.3 ± 16.0	23.8 ± 25.0	<0.001
Female sex – no. (%)	47 (42.7)	30 (27.3)	17 (15.5)	0.970
Height in cm	123.2 ± 36.6	114.6 ± 33.1	138.26 ± 38.0	<0.001
Weight in kg	33.2 ± 27.1	27.0 ± 22.8	44.5 ± 30.9	<0.001

Ψ Plus-minus values are means ± SD. There were 110 patients in the study. The baseline score was collected preoperatively.

With respect to operative characteristics, there were significant differences in the operative times between the two cohorts, when analysed using Welch's *T*-Test and two-tailed test for continuous variables. Patients whose defects were corrected using the baffle for reconstruction of the superior vena cava had average total operative time of 209 min (SD ± 57.7) with an average time on cardiopulmonary bypass of 96.1 min (SD ± 34.9) and an average cross clamp time of 46 min (SD ± 23.1). The average times for patients undergoing superior vena cava correction using the “Z” flap technique were as follows: total operative time of 182.6 min (SD ± 45, *p* < 0.05), CPB duration of 71.4 min (SD ± 27.3, *p* < 0.05) and an average cross clamp time of 35.7 min (SD ± 17, *p* < 0.05). Operative characteristics have been summarized in Table 2.

**Table 2:** Operative characteristics of patients undergoing sinus venosus atrial septal defect^Ψ^.

Characteristic	Total	Correction by baffle	Correction by “Z” Flap.	*p* value
Mean operative time (min.)	199.5 ± 54.8	209.2 ± 57.7	182.6 ± 45	<0.05
Mean time on CPB (min.)	87.1 ± 34.3	96.1 ± 34.9	71.3 ± 27.26	<0.001
Mean cross clamp time (min.)	42.2 ± 21.6	46 ± 23.1	35.7 ± 17	<0.05

CPB, cardiopulmonary bypass.

Ψ Plus-minus values mean ± SD.

It is worth noting that there was no significant stenosis of the superior vena cava demonstrable via echocardiography. There were however a total of 7 cases who had a residual shunt across the corrected atrial septal defect. 2 of these patients were operated upon at a later date. There was however no statistically significant residual shunt upon conducting a Fisher's exact test.

On further analysis of the postoperative occurrence of rhythm disturbances, we found that a total of 60 patients (54.5%) developed postoperative rhythm disturbances. On further subdivision, 10 patients (9.1%) developed a sinoatrial block with an atrio-ventricular junctional rhythm. The most common form of rhythm disturbance, with 45 patients (40.1%) developing this was a right bundle branch block. 5 patients (4.5%) developed postoperative atrial fibrillation. There were no cases requiring the implantation of a permanent pacemaker.

On further analysis of the cohorts, the singular statistically significant difference was seen in the development of the atrio-ventricular junctional rhythm. 7 patients (17.5%) undergoing correction using the “Z” flap group developed an atrio-ventricular junctional rhythm and only 3 patients (4.3%) undergoing the baffle correction were subject to this. With a *p* value of 0.035 (*p* < 0,05) and an OR of approx. 4.47, there is a significantly higher incidence of the development of atrio-ventricular junctional rhythm in patients undergoing correction of a Sinus venosus atrial septal defect (SVASD) using the “Z” flap method.

Further subgroup analysis of rhythm disturbances did not yield a statistically significant difference between the two groups and has been summarized in Table 3.

**Table 3:** Postoperative rhythm disturbances.

Rhythm disturbance	Total	Correction by baffle	Correction by. “Z” flap.	*p* value
Atrio-ventricular junctional rhythm	10 (9.1%)	3	7 (OR = 4.74)	<0.05
RBBB	45 (40.1%)	30 (OR = 0.8)	15	0.6877
Atrial fibrillation	5 (4.5%)	2	3 (OR = 0.36)	0.351
Sum	60	35	25	0.2361

Values indicate individual patients. Significant *p*-value (*p* < 0.05). OR, odds ratio; RBBB, right bundle branch block.

## Discussion

Sinus venosus atrial septal defect (SVASD) is a distinct form of interatrial communication characterized by abnormal incorporation of the caval veins into the atrial septum and is associated with partial anomalous pulmonary venous return (PAPVR) (1). There are two anatomical variants which are recognized: the true sinus venosus defect, defined by a deficiency of the atrial septum adjacent to the superior or inferior vena cava, and the false sinus venosus defect, in which the atrial septum is anatomically intact but overridden by anomalous pulmonary venous drainage into the caval vein or right atrium (1, 2). Both entities regularly result in significant left-to-right shunting and chronic right ventricular volume overload (3). A major anatomical challenge for surgical correction, is the close spatial relationship between the anomalous pulmonary veins and the sinus node region (4). The American Heart Association (AHA) guidelines emphasize these anatomical considerations, recommending a surgical repair in the presence of right heart dilation regardless of occurrence of any symptoms (5).

Two principal surgical techniques are currently employed for SVASD correction (6). The conventional single-patch technique, which involves intracardiac redirection of pulmonary venous flow to the left atrium using a prosthetic or autologous pericardial patch sutured along the atrial septal margin and caval vein wall (6, 7). In cases where the superior vena cava (SVC) lumen is compromised, an additional patch or caval augmentation may be required to prevent any postoperative stenosis (7). In contrast, the double-patch technique consists of two distinct reconstructions: one patch redirects pulmonary venous blood to the left atrium, while a second patch enlarges the caval vein to maintain unobstructed systemic venous return (8). An anatomical variant of the double-patch technique uses mobilized native atrial tissue to form the caval baffle, thereby minimizing the use of foreign material (9). The two mentioned techniques use substantially different operative steps, where in the conventional approach, a precise sizing and positioning of the intracardiac patch are critical to avoid pulmonary venous obstruction or SVC narrowing (6–8). Moreover when native atrial tissue is used as part of the reconstruction, the atrial wall is incised and re-directed to create a physiological baffle, preserving tissue continuity and compliance (9, 10).

Prolonged ischemic time is a critical driver of postoperative morbidity in congenital cardiac surgery (11). In addition, extended aortic cross-clamp and cardiopulmonary bypass durations are associated with increased myocardial ischemic reperfusion injury, impaired ventricular function, and higher inflammatory response (11, 12). Ischemic myocardial injury can lead to transient or permanent ventricular dysfunction, necessitating prolonged inotropic support and increasing intensive care unit (ICU) length of stay (12). Additionally, prolonged ischemic time has been linked to a higher incidence of acute kidney injury (AKI), likely mediated by systemic inflammation and reduced renal perfusion and neurologically a longer bypass time can increase the risk of cerebral hypoperfusion and embolic events (13, 14).

An indirect protective effect may be observed through techniques that streamline a reconstruction, which at the same time reduce the operative complexity (15). The double-patch technique, when performed efficiently, may reduce repeated patch revisions and limit ischemic exposure as well as sparing prosthetic material, which may decrease inflammatory activation and foreign body reaction (8, 16). In addition, an autologous tissue integration promotes endothelialization and may reduce long term risks of fibrosis or stenosis at the reconstruction site (17).

The use of native atrial anatomy in double-patch or anatomical reconstruction techniques offers additional physiological advantages (9). Autologous tissue demonstrates superior compliance as mentioned earlier compared with synthetic materials, which may reduce flow turbulence and shear stress (17). Maintenance of atrial anatomy and original architecture may protect the sinus node, potentially leading to decreasing occurrence of postoperative AV junctional rhythms (18). These considerations align with AHA recommendations favoring anatomical restoration and avoidance of venous obstruction, however, both surgical techniques are considered valid and effective according to contemporary AHA and ACC guidelines (5). Operative mortality for SVASD repair in modern series is low, typically below 1%–2%, irrespective of technique (19). Long-term survival is excellent and largely determined by preoperative right heart remodeling and associated anomalies rather than surgical strategy alone (20). The conventional single patch approach is known to be widely reproducible and suitable for most anatomical configurations (6). The double-patch technique, while technically more demanding, may provide superior anatomical correction in cases with high pulmonary venous insertion or significant caval overrides (8). Atrial arrhythmias, particularly atrial fibrillation, remain a relevant late complication after SVASD repair (18). The similar rate of incidence of right bundle branch block (RBBB) in both cohorts, suggests that factors other than operative technique (e.g. right ventricular function) are responsible for this occurrence. Any surgical intervention and manipulation near the sinus node region and atrial conduction pathways heightens the susceptibility to postoperative arrhythmic occurrences, such as atrial fibrillation, which in particular is associated with occurrence of thromboembolic events, including ischemic stroke and transient ischemic attack (18, 21, 22). The occurrence of postoperative AKI may further amplify cardiovascular risk through systemic inflammation and endothelial dysfunction (13). Therefore, strategies that reduce ischemic time and preserve native tissue may therefore have indirect benefits on rhythm stability and end-organ protection (15).

Over a weighted mean follow-up of 8.6 years, the pooled incidence of pacemaker insertion following surgical sinus venosus ASD repair was 2% (1%–2%), and the pooled incidence of sinus node dysfunction was 4% (2%–6%). This dissociation between the incidence of detectable junctional rhythm and the rate of pacemaker implantation reflects the predominantly benign yet persistent nature of these rhythm disturbances (23).

In summary, both the conventional patch repair and the double-patch anatomical reconstruction are guideline supported and effective surgical options for sinus venosus defect correction (5). The double-patch technique offers potential advantages in anatomical fidelity, material sparing, and flow dynamics, particularly when native tissue is utilized (9). The conventional technique remains a robust and reliable approach with excellent outcomes in experienced hands (6). Individualized surgical planning based on detailed anatomical assessment and institutional expertise remains essential to optimize outcomes and minimize long-term complications (5).

## Conclusions

Both methods used for the correction of sinus venosus atrial septal defect have overall comparable incidence of rhythm disturbances. Each method contains inherent advantages and disadvantages. Correction using the “Z” flap technique appears to be swifter, but has a significantly higher risk of development of a sinoatrial block.

## Data Availability

The datasets supporting the conclusions of this article will be made available by the authors upon request. Inquiries can be directed to the corresponding author.
